# Robustness of Automated Methods for Brain Volume Measurements across Different MRI Field Strengths

**DOI:** 10.1371/journal.pone.0165719

**Published:** 2016-10-31

**Authors:** Rutger Heinen, Willem H. Bouvy, Adrienne M. Mendrik, Max A. Viergever, Geert Jan Biessels, Jeroen de Bresser

**Affiliations:** 1 Department of Neurology and Neurosurgery, Brain Center Rudolph Magnus, University Medical Center Utrecht, Utrecht, the Netherlands; 2 Image Sciences Institute, University Medical Center Utrecht, Utrecht, the Netherlands; 3 Department of Radiology, University Medical Center Utrecht, Utrecht, the Netherlands; Generalitat Valenciana, SPAIN

## Abstract

**Introduction:**

Pooling of multicenter brain imaging data is a trend in studies on ageing related brain diseases. This poses challenges to MR-based brain segmentation. The performance across different field strengths of three widely used automated methods for brain volume measurements was assessed in the present study.

**Methods:**

Ten subjects (mean age: 64 years) were scanned on 1.5T and 3T MRI on the same day. We determined robustness across field strength (i.e., whether measured volumes between 3T and 1.5T scans in the same subjects were similar) for SPM12, Freesurfer 5.3.0 and FSL 5.0.7. As a frame of reference, 3T MRI scans from 20 additional subjects (mean age: 71 years) were segmented manually to determine accuracy of the methods (i.e., whether measured volumes corresponded with expert-defined volumes).

**Results:**

Total brain volume (TBV) measurements were robust across field strength for Freesurfer and FSL (mean absolute difference as % of mean volume ≤ 1%), but less so for SPM (4%). Gray matter (GM) and white matter (WM) volume measurements were robust for Freesurfer (1%; 2%) and FSL (2%; 3%) but less so for SPM (5%; 4%). For intracranial volume (ICV), SPM was more robust (2%) than FSL (3%) and Freesurfer (9%). TBV measurements were accurate for SPM and FSL, but less so for Freesurfer. For GM volume, SPM was accurate, but accuracy was lower for Freesurfer and FSL. For WM volume, Freesurfer was accurate, but SPM and FSL were less accurate. For ICV, FSL was accurate, while SPM and Freesurfer were less accurate.

**Conclusion:**

Brain volumes and ICV could be measured quite robustly in scans acquired at different field strengths, but performance of the methods varied depending on the assessed compartment (e.g., TBV or ICV). Selection of an appropriate method in multicenter brain imaging studies therefore depends on the compartment of interest.

## Introduction

Pooling of multicenter brain MRI data is a trend in various research fields, for example in studies on ageing related brain diseases. [1–3] Pooling of multicenter data increases sample size (and thus statistical power) and can support a faster patient inclusion. Moreover, findings of multicenter studies may have larger external validity and are more readily translatable to a clinical setting. However, use of different MRI acquisition techniques, for example with regard to scanner types or field strength [4–6], across centers could introduce variation in results of frequently used MR-based automated brain segmentation methods. [6] This variation could potentially even be larger than the actual effect size of the brain changes studied. [7,8]

To date, the performance of the most recent versions of Statistical Parametric Mapping (SPM) [9], Freesurfer [10] and FMRIB Software Library (FSL) [11]) in datasets with different MRI acquisition techniques (such as different field strengths) is not well studied. Performance of these methods can be assessed in terms of robustness (i.e., whether measured volumes on scans with different acquisitions techniques in the same subjects are similar) and accuracy (i.e., whether measured volumes correspond with expert-defined reference volumes). It is important to consider both measures of performance together, since neither a robust, inaccurate method nor an accurate, non-robust method does not lead to valid results in a multicenter study.

In the present study, we evaluated the performance of three widely used automated methods for brain volume measurements (SPM, Freesurfer and FSL). Robustness was assessed in subjects that were scanned on 1.5T and 3T MRI on the same day. Accuracy was determined by comparing the measurements of the methods with manual segmentations on 3T MRI scans of additional subjects.

## Materials and Methods

### Automated methods for brain volume measurements and image processing

SPM (version 12), Freesurfer (version 5.3.0) and FSL (version 5.0.7 with use of SIENAX, version 2.6) were used to calculate brain volumes and intracranial volume (ICV) on T1-weighted MRI images.

#### SPM12

SPM (Wellcome Department of Cognitive Neurology, Institute of Neurology, Queen Square, London; available at http://www.fil.ion.ucl.ac.uk/spm/) uses the unified segmentation (US) algorithm, which combines tissue classification, bias correction and image registration in the same generative model. [9] It produces partial volume segmentation results for each tissue compartment, using tissue prior probability maps based on intensity values. From these results absolute volumes of gray matter (GM), white matter (WM) and cerebrospinal fluid (CSF) are calculated. Additional tissue maps for soft tissue, bone and air/background were included in SPM8 and are now part of standard segmentation. [12] This reduces the possibility of misclassification of non-brain tissue. In our study, segmentation was performed using the advised default settings. Partial volume segmentation results for each of the three tissue compartments (GM, WM and CSF) were obtained and extracted by using the ‘Tissue Volumes’ utility in SPM. Total brain volume (TBV) was calculated by summing up GM and WM volumes. ICV was determined by summing up TBV and CSF volumes.

#### Freesurfer

Freesurfer (Martinos Center for Biomedical Imaging, Harvard-MIT, Boston; available at http://surfer.nmr.mgh.harvard.edu/) consists of surface based analysis [13] and volumetric segmentation. [10,14] It involves intensity non-uniformity correction [15], affine transformation to a MNI305 template, intensity normalization, removal of non-brain tissue [16], linear and non-linear transformations to a probabilistic brain atlas and labeling of cortical and subcortical structures. [10,14] It uses a Markov Random Field model for each structure for each point in space. Spatial localization priors are used in determining the right label per voxel. [17] Since Freesurfer version 5.2, surface-based calculations are used to calculate various brain volumes to get better accuracy. In our study, segmentation was performed using default settings (i.e. using the command: ‘recon-all’). For our study, we used the compartment measurements reported by Freesurfer. All volumes were extracted from the stat files that Freesurfer produces using the ‘asegstats2table’ command. Since Freesurfer estimates ICV and does not perform segmentation of extracerebral CSF, we obtained the CSF volume by subtracting TBV from the estimated ICV.

#### FSL

FSL (Analysis Group, FMRIB, Oxford, United Kingdom; available at http://fsl.fmrib.ox.ac.uk/fsl/fslwiki/) uses the SIENAX package for estimating brain tissue volumes from a single image. [11,18,19] SIENAX starts by extracting brain and skull images from the single whole-head input data. [20] The brain image is then affine-registered to MNI152 space. [21,22] Next, tissue-type segmentation with partial volume estimation is carried out. [23] From these estimations, GM, WM and ventricular CSF volumes were calculated. In our study, we stripped excessive slices at the level of the neck to allow accurate skull stripping, which in an earlier study yielded optimal results with various scan protocols. [24] In concordance with a previous study investigating the optimal settings for the brain extraction tool (BET) we used the following settings: a fraction intensity threshold value of 0.1 and use of the B-option (bias field and neck clean up). [24] Partial volume segmentation results for each of the three tissue compartments (GM, WM and CSF) were obtained. TBV was calculated by summing up GM and WM volumes (as reported by FSL). We used MeVisLab (MeVis Medical Solutions AG, Bremen, Germany, version 2.5) to obtain CSF measurements from the FSL partial volume segmentation (since FSL only reports ventricular CSF volume). ICV was calculated by summing up GM, WM and (total) CSF volumes.

### Robustness analysis

#### Study population

To determine the robustness across field strengths, subjects were scanned on 1.5T and 3T MRI on the same day. The intention was to recruit a group of patients with ageing related brain changes, but without a known primary cerebral disease. Therefore, patients, aged 50–80 years, with chronic idiopathic axonal polyneuropathy (CIAP) were recruited from an ongoing cohort study at the University Medical Center Utrecht, Utrecht, the Netherlands between September 2012 and October 2013. [25] Exclusion criteria were a history of brain disease, not living independently and/or a contra-indication for MRI. Written informed consent was provided by all participants. The study was approved by the local medical ethics committee.

#### MRI data acquisition

The 1.5T MRI (Achieva; Philips, Best, the Netherlands) protocol consisted of the following sequences covering the entire brain: a sagittal 3D T1-weighted sequence (170 continuous slices, voxel size: 0.94x0.94x1.00 mm^3^, repetition time (TR)/echo time (TE): 7.0/3.2 ms) and an axial 2D fluid attenuated inversion recovery (FLAIR) sequence (38 continuous slices, voxel size: 0.90x0.90x4.0 mm^3^, TR/TE/inversion time (TI): 6.000/100/2000 ms). The 3T MRI (Achieva; Philips, Best, the Netherlands) protocol consisted of the following sequences: a sagittal 3D T1-weighted sequence (192 continuous slices, voxel size: 1.00x1.00x1.00 mm^3^, TR/TE: 7.9/4.5 ms) and an axial 2D FLAIR sequence (48 continuous slices, voxel size: 0.96x0.95x3.00 mm^3^, TR/TE/TI: 11000/125/2800 ms). Additionally, to evaluate robustness across different spatial resolutions (high versus low), the 3D T1 images of the 1.5T and 3T MRI scans were downsampled to a voxel size of 0.96x0.96x3.00 mm^3^.

#### Statistical analysis

Non-parametric statistical tests were used because of the limited number of subjects. Robustness was assessed in two ways. First, we assessed potential systematic bias across field strength for each method with a Wilcoxon signed rank test. Next, the amount of variation/bias between 3T and 1.5T measurements was assessed by mean absolute differences (also expressed as a percentage of the mean volume at 3T). To further evaluate these differences we determined coefficients of repeatability as well as Bland Altman plots. The coefficient of repeatability is calculated by multiplying the standard deviation of the absolute differences (i.e., square root of the mean squared difference) between measurements at 3T and 1.5T by 1.96. [26]. It thus represents the upper limit of the mean difference between two measurements in 95% of cases. Bland Altman plots give a graphical representation of presence/absence of systematic bias and the amount of variation between measurements. In these plots, a mean difference close to zero indicates absence of systematic bias. A narrow width of the limits of agreement reflects a small amount of variation between measurements at 3T and 1.5T.

In secondary analyses we repeated the entire analysis for the high versus low resolution comparison.

### Accuracy analysis

#### Study population and MRI data

To determine accuracy, scans from healthy control subjects were selected from a cohort study of functionally independent elderly subjects (65–80 years of age) without a history of stroke or other brain diseases. [27] Subjects were scanned on 3T MRI with an identical scanning protocol as the subjects of the robustness analysis. Written informed consent was provided by all participants and the study was approved by the local medical ethics committee.

#### Reference data

Manual segmentations were used as reference data. The procedure for manual segmentations was described previously (for details see [28]). First, the 3D T1 and 2D T1-IR scans were registered to the 2D FLAIR scan by means of Elastix. [29] The 3D T1 scan was downsampled so that all scans had a resulting voxel size of 0.96x0.96x3.00 mm^3^. Subsequently, bias correction was performed using SPM8. [30] Manual segmentations were performed on the axial T1, T1-IR and FLAIR slices by trained research assistants, using an in-house developed tool based on MeVisLab (MeVis Medical Solutions AG, Bremen, Germany). This tool allowed a closed freehand spline drawing technique, which was used to delineate the outline of each tissue compartment (GM, WM and CSF). The closed contours were then converted into hard segmentations. The resulting images were checked and corrected by three experts (WB, AM, JdB).

Because manual segmentations that separate the cerebellum in GM and WM cannot be performed with high reliability, we chose not to differentiate between GM and WM in the manual segmentations of the cerebellum and other infratentorial structures. A mask of the manually segmented infratentorial structures was used to obtain supratentorial GM and WM volumes for each of the three automated segmentation methods for the analysis of accuracy in MeVisLab (MeVis Medical Solutions AG, Bremen, Germany, version 2.5). In the accuracy analysis, the infratentorial structures were not excluded from the TBV, CSF volume and ICV for all methods.

#### Statistical analysis

Non-parametric statistical tests were used because of the limited number of subjects. We performed similar analyses as for the robustness part, but now volume measurements of the methods were compared with the reference standard. Furthermore, we also calculated a Dice’s similarity coefficient (DSC) to evaluate spatial overlap between the segmentations of the methods and the reference data. As required for these analyses, the probabilistic segmentations of SPM and FSL were thresholded on a probability of 0.5. For Freesurfer the spatial overlap analyses required the output to be brought to native space by nearest neighbor interpolation (using the following command: ‘mri_vol2vol—mov aseg.mgz—targ rawavg.mgz—regheader—o asegCorrect.mgz—nearest—no-save-reg’) and divided in the three tissue compartments (GM, WM, CSF).

## Results

### Quality assessment

**Fig 1:**
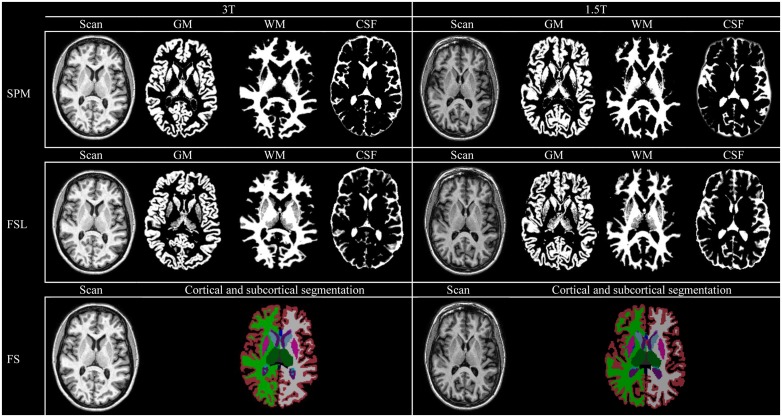
Automated brain volume measurements at 3T and 1.5T. Examples of the performed measurements for the robustness analysis for one subject on 3T and 1.5T MRI. Individual measurements are shown using a probabilistic (SPM and FSL) or a binary approach (Freesurfer). GM: gray matter. WM: white matter. CSF: cerebrospinal fluid.

**Fig 2:**
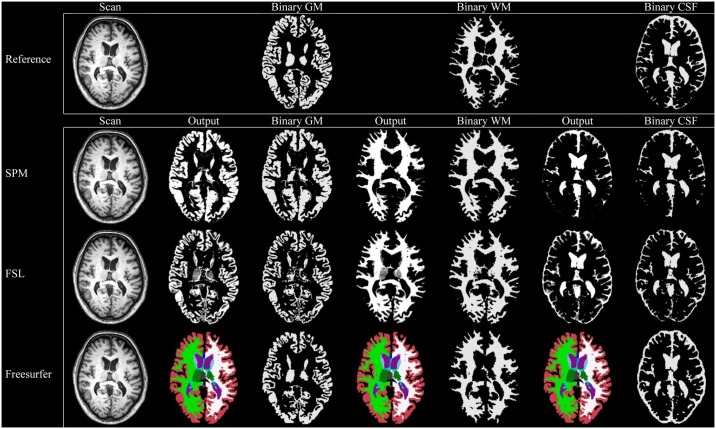
Reference measurements and automated brain volume measurements at 3T. Examples of the performed measurements for the accuracy analysis for one subject on 3T MRI. Individual measurements are shown in native space (probabilistic for SPM and FSL, and binary for Freesurfer). Also, binary measurements for each tissue compartment are shown for all methods (using a threshold of 0.5 in case of probabilistic segmentation). GM: gray matter. WM: white matter. CSF: cerebrospinal fluid.

Examples of the performed measurements of one subject using SPM, Freesurfer and FSL are shown in Fig 1 for the robustness analysis and in Fig 2 for the accuracy analysis. Output of all subjects was visually checked and was considered to be of good quality. No manual editing was performed. None of the patients proved to have (major) structural abnormalities on their scans that could influence automated segmentation results. Minor segmentation differences between methods can visually be appreciated in the figure. For example FSL generally segments less GM in the basal ganglia and thalamus, while this was less pronounced in SPM (Figs 1 and 2).

### Robustness across field strength

Ten patients (four male, six female) were included for the robustness analyses. They had a mean age of 64 ± 7 years. For the evaluation of robustness across 3T and 1.5T, mean and individual brain volume measurements are presented in Table 1 and S1 Fig.

**Table 1:** Automated volume measurements at 3T and 1.5T (n = 10).

			SPM	Freesurfer	FSL
**TBV**	*1*.*5T*	*3D*	1062 ± 80	1016 ± 72	1042 ± 74
	*3T*	*3D*	1020 ± 57	1013 ± 67	1049 ± 82
**GM**	*1*.*5T*	*3D*	610 ± 46	548 ± 38	552 ± 41
	*3T*	*3D*	590 ± 29	545 ± 37	554 ± 43
**WM**	*1*.*5T*	*3D*	452 ± 38	468 ± 38	491 ± 39
	*3T*	*3D*	430 ± 33	468 ± 36	495 ± 42
**CSF**	*1*.*5T*	*3D*	294 ± 56	440 ± 72	333 ± 36
	*3T*	*3D*	323 ± 90	342 ± 118	364 ± 43
**ICV**	*1*.*5T*	*3D*	1356 ± 121	1456 ± 102	1376 ± 99
	*3T*	*3D*	1343 ± 128	1355 ± 168	1413 ± 111

Note: All volumes are expressed as means (in cc) ± SD. TBV: total brain volume. GM: gray matter volume. WM: white matter volume. CSF: cerebrospinal fluid volume. ICV: intracranial volume.

#### TBV

**Fig 3:**
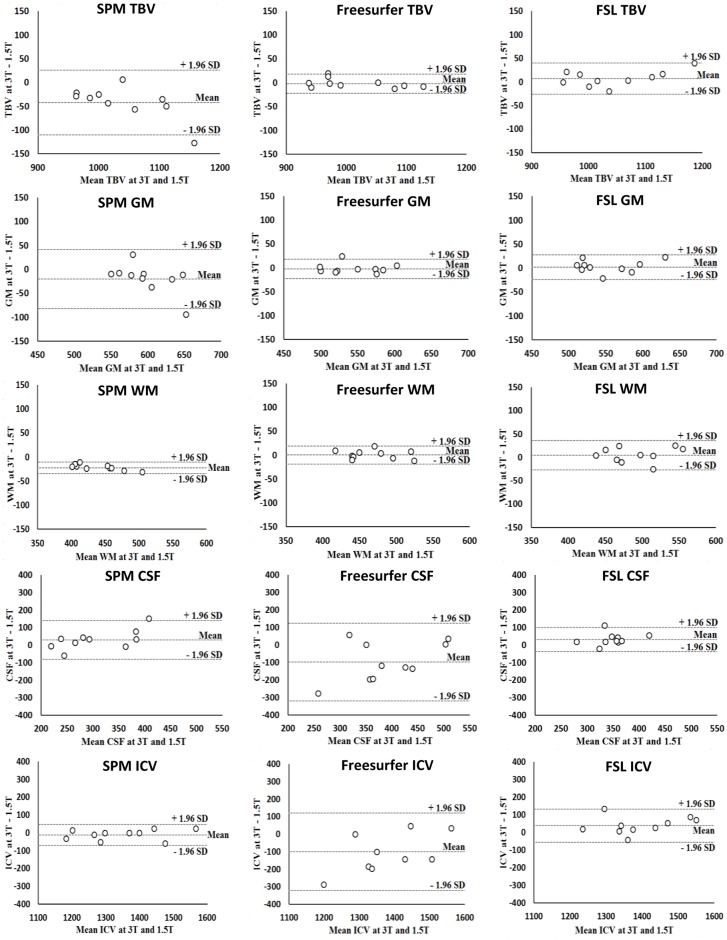
Bland-Altman plots at 3T and 1.5T. X-axis: mean brain volume measurement at 3T and 1.5T. Y-axis: difference (in cc) in brain volume measurement between 3T and 1.5T. The mean, lower (- 1.96 SD) and upper (+ 1.96 SD) limits of agreement are shown. A negative difference on the y-axis is seen when brain volume measurements at 1.5T were larger than at 3T. TBV: total brain volume. GM: gray matter volume. WM: white matter volume. CSF: cerebrospinal fluid volume. ICV: intracranial volume.

Measurements of TBV at 3T and 1.5T were robust for Freesurfer and FSL (i.e., non-significant mean differences between field strengths as shown in Table 2; see also the Bland Altman plots in Fig 3). Mean absolute differences were also small. Freesurfer gave a mean absolute difference ± SD of 8.4 ± 5.6 cc, which is <1% of mean TBV as measured by this method at 3T. Corresponding figures for FSL were 14 ± 12 cc; 1%. The coefficients of repeatability were in line with these findings (see Fig 3). By comparison, SPM was less robust across field strength for TBV (mean difference ± SD: -42 ± 33 cc; *p* = 0.007; see also the Bland Altman plots in Fig 3). The mean absolute difference (43 ± 33 cc; 4%) was also larger than that of the other methods.

**Table 2:** Robustness analysis across different field strengths (n = 10).

			SPM	Freesurfer	FSL
**TBV**	*3T vs 1*.*5T (3D)*	*Mean difference*	-42 ± 35*	-2 ± 10	7 ± 17
		*Mean absolute difference*	43 ± 33	8 ± 6	14 ± 12
		*as % of mean TBV at 3T*	4	<1	1
		*Coefficient of repeatability*	107	20	35
		*as % of mean TBV at 3T*	11	2	3
**GM**	*3T vs 1*.*5T (3D)*	*Mean difference*	-20 ± 32*	-2 ± 10	2 ± 13
		*Mean absolute difference*	26 ± 26	8 ± 6	10 ± 8
		*as % of mean GM at 3T*	5	1	2
		*Coefficient of repeatability*	72	20	25
		*as % of mean GM at 3T*	13	4	5
**WM**	*3T vs 1*.*5T (3D)*	*Mean difference*	-22 ± 6*	<1 ± 10	4 ± 16
		*Mean absolute difference*	22 ± 6	8 ± 5	13 ± 9
		*as % of mean WM at 3T*	4	2	3
		*Coefficient of repeatability*	46	18	32
		*as % of mean WM at 3T*	11	4	6
**CSF**	*3T vs 1*.*5T (3D)*	*Mean difference*	29 ± 56	-98 ± 113	31 ± 35*
		*Mean absolute difference*	45 ± 43	115 ± 93	36 ± 29
		*as % of mean CSF at 3T*	14	34	10
		*Coefficient of repeatability*	122	291	90
		*as % of mean CSF at 3T*	36	85	25
**ICV**	*3T vs 1*.*5T (3D)*	*Mean difference*	-13 ± 30	-100 ± 113*	38 ± 48*
		*Mean absolute difference*	23 ± 21	115 ± 95	46 ± 39
		*as % of mean ICV at 3T*	2	9	3
		*Coefficient of repeatability*	62	293	118
		*as % of mean ICV at 3T*	5	22	8

Note: All volumes (in cc) are expressed as means ± SD. Coefficients of repeatability are expressed as a volume (in cc). TBV = total brain volume. GM: gray matter volume. WM: white matter volume. CSF: cerebrospinal fluid volume. ICV: intracranial volume. Mean differences between 3T and 1.5T were tested for each method separately using Wilcoxon signed rank test (* *p*<0.05).

#### GM volume

Freesurfer and FSL were robust for GM volume measurements across field strength (Table 2 and the Bland Altman plots in Fig 3) and the mean absolute differences were small: Freesurfer (8 ± 6 cc; 1%); FSL (10 ± 8 cc; 2%). SPM was less robust for GM volume across field strength (-20 ± 32 cc; *p* = 0.047; see also the Bland Altman plots in Fig 3). In line with this, the mean absolute difference (26 ± 26 cc; 5%) was larger compared with Freesurfer and FSL (Table 2; see also the coefficient of repeatability in Table 2).

#### WM volume

WM volume measurements were robust across field strengths for Freesurfer and FSL (Table 2 and the Bland Altman plots in Fig 3) and the mean absolute differences were small: Freesurfer (8 ± 5 cc; 2%); FSL (13 ± 9 cc; 3%). For SPM, WM volume was less robust across field strength (-22 ± 6 cc; *p =* 0.005). The mean absolute difference (22 ± 6 cc; 4%) was also larger than for the other methods (Table 2).

#### CSF volume

None of the methods was robust for CSF. Substantial relative and absolute differences in measured CSF volume across field strength were observed for all methods (Table 2, Fig 3), which was also reflected in a large coefficient of repeatability (Table 2).

#### ICV

ICV measurements were robust across field strengths for SPM (Table 2 and the Bland Altman plots in Fig 3) with also a small mean absolute difference (23 ± 21 cc; 2%). ICV measurements were less robust across field strength for Freesurfer (-100 ± 113 cc; *p* = 0.037) and FSL (38 ± 48 cc; *p* = 0.028; see also the Bland Altman plots in Fig 3). The mean absolute difference was smaller for FSL (47 ± 39 cc; 3%) than for Freesurfer (115 ± 95 cc; 9%); which was reflected in the coefficient of repeatability (Table 2).

### Robustness across different spatial resolutions

In secondary analyses we assessed robustness across different spatial resolutions (high versus low, i.e., 3T 3D T1 versus downsampled T1). Mean brain volume measurements at 3D and downsampled resolutions are shown in S1 Table and measurements per subject are shown in S2 Fig. For SPM and FSL, results were comparable with the across field strength analysis (see S2 Table). The performance of Freesurfer was less robust for TBV (18 ± 9 cc; *p* = 0.005), GM (25 ± 9 cc; *p* = 0.005) and WM (-6 ± 5 cc; *p* = 0.013; see also Bland Altman plots in S2 Fig) when using low resolution T1-weighted MR-images for segmentation. The mean absolute differences for Freesurfer (as % of mean volume at 3D for TBV, GM and WM: 2%; 5%; 1%) were also larger compared with the 3T versus 1.5T comparison of Freesurfer (1%; 1%; 1%). The other results for Freesurfer were in line with the results of the 3T versus 1.5T comparison.

### Accuracy assessments

Twenty subjects (ten male, ten female) were included for the accuracy analysis. They had a mean age of 71 ± 4 years. For the comparison between the automated methods and manual segmentation, individual brain volume measurements are presented in S4 and S5 Figs.

#### TBV

**Fig 4:**
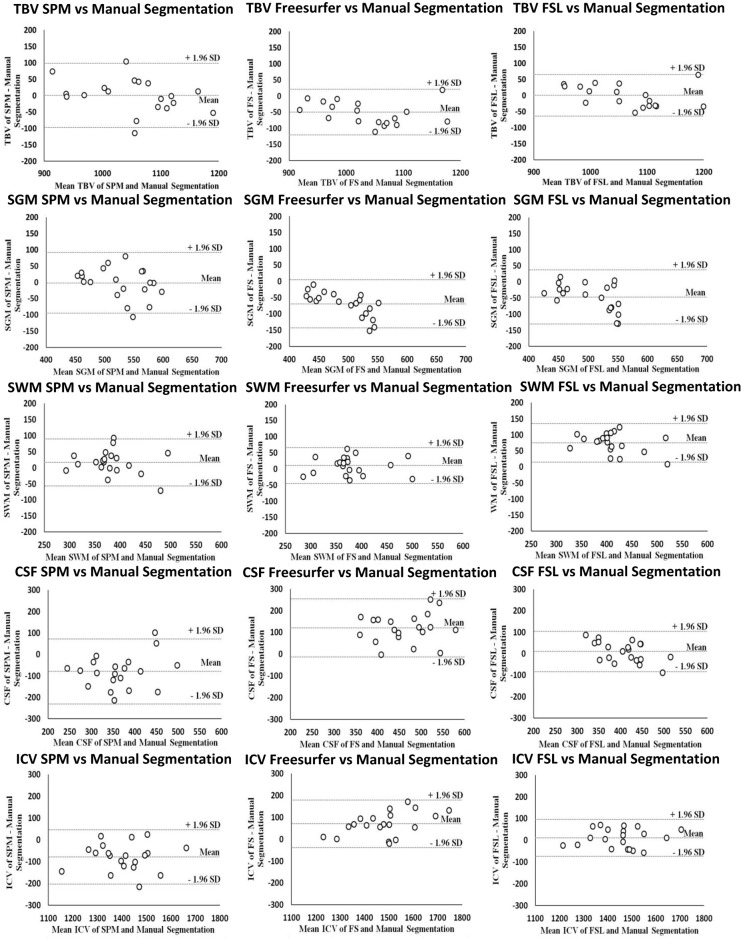
Bland-Altman plots automated versus manual volume measurements. X-axis: mean brain volume measurement of automated and manual volume measurements. Y-axis: difference (in cc) in brain volume measurement between automated and manual volume measurements. The mean, lower (- 1.96 SD) and upper (+ 1.96 SD) limits of agreement are shown. A negative difference on the y-axis is seen when brain volume measurements were larger with manual volume measurements than with automated volume measurements. TBV: total brain volume. GM: gray matter volume. WM: white matter volume. CSF: cerebrospinal fluid volume. ICV: intracranial volume.

Measurements of TBV were accurate compared to manual segmentation for FSL and SPM (non-significant mean differences as shown in Table 3; see also the Bland Altman plots in Fig 4) with small mean absolute differences: FSL, when compared to mean TBV as measured by manual segmentation (29 ± 15 cc; 3%); SPM (36 ± 34 cc; 3%). Freesurfer was less accurate in measuring TBV (mean difference: -50 ± 36 cc; *p*<0.001; also see the Bland Altman Plots in Fig 4). The mean absolute difference (52 ± 32 cc; 5%) was also larger for Freesurfer than for to the other methods.

**Table 3:** Accuracy of automated volume measurements (n = 20).

		SPM	Freesurfer	FSL
**TBV**	*Volume (method)*	1045 ± 82	994 ± 85	1044 ± 85
	*Volume (manual segmentation)*	1044 ± 97	1044 ± 97	1044 ± 97
	*Mean difference*	1 ± 50	-50 ± 36*	<1 ± 33
	*Mean absolute difference*	36 ± 34	52 ± 32	29 ± 15
	*as % of mean TBV with manual segmentation*	3	5	3
	*Dice’s similarity coefficient*	0.94 ± 0.008	0.92 ± 0.007	0.94 ± 0.006
**SGM**	*Volume (method)*	526 ± 44	459 ± 33	481 ± 38
	*Volume (manual segmentation)*	528 ± 59	528 ± 59	528 ± 59
	*Mean difference*	-2 ± 47	-69 ± 38*	-47 ± 43*
	*Mean absolute difference*	35 ± 31	69 ± 38	49 ± 40
	*as % of mean SGM with manual segmentation*	7	13	9
	*Dice’s similarity coefficient*	0.79 ± 0.03	0.77 ± 0.02	0.77 ± 0.03
**SWM**	*Volume (method)*	390 ± 50	384 ± 56	448 ± 46
	*Volume (manual segmentation)*	373 ± 56	373 ± 56	373 ± 56
	*Mean difference*	17 ± 37*	11 ± 28	75 ± 31*
	*Mean absolute difference*	31 ± 26	26 ± 15	75 ± 31
	*as % of mean SWM with manual segmentation*	8	7	20
	*Dice’s similarity coefficient*	0.85 ± 0.03	0.85 ± 0.02	0.84 ± 0.03
**CSF**	*Volume (method)*	324 ± 83	523 ± 77	413 ± 45
	*Volume (manual segmentation)*	403 ± 68	403 ± 68	403 ± 68
	*Mean difference*	-80 ± 77*	120 ± 68*	10 ± 48
	*Mean absolute difference*	95 ± 56	120 ± 68	42 ± 23
	*as % of mean CSF with manual segmentation*	24	30	10
	*Dice’s similarity coefficient*	0.69 ± 0.05	0.65 ± 0.03	0.70 ± 0.03
**ICV**	*Volume (method)*	1368 ± 119	1517 ± 144	1458 ± 121
	*Volume (manual segmentation)*	1447 ± 118	1447 ± 118	1447 ± 118
	*Mean difference*	-78 ± 63*	70 ± 55*	11 ± 43
	*Mean absolute difference*	84 ± 55	74 ± 50	39 ± 21
	*as % of mean ICV with manual segmentation*	6	5	3
	*Dice’s similarity coefficient*	0.95 ± 0.01	0.93 ± 0.007	0.95 ± 0.007

Note: All volumes are expressed as means (in cc) ± SD. The DSC is shown as mean ± SD. TBV = total brain volume (sum of supratentorial GM and WM, cerebellar and brainstem volume); SGM = supratentorial GM volume; SWM = supratentorial WM volume; CSF = total cerebrospinal fluid volume; ICV = intracranial volume. Differences between automated and manual measurements were tested for each method separately using Wilcoxon signed rank test (* *p*<0.05).

#### GM volume

SPM was accurate for supratentorial GM volume, but FSL (-47 ± 43 cc; *p*<0.001) and Freesurfer (-69 ± 38 cc; *p*<0.001) were less accurate (Table 3 and the Bland Altman plots in Fig 4). The mean absolute difference was also smaller for SPM (35 ± 31 cc; 7%) than for FSL (49 ± 40 cc; 9%) and Freesurfer (69 ± 38 cc; 13%).

#### WM volume

Supratentorial WM volume measurements for Freesurfer were accurate (Table 3 and the Bland Altman plots in Fig 4). The mean absolute difference (26 ± 15 cc; 7%) was also smaller for Freesurfer than for the other methods. SPM (17 ± 37 cc; *p* = 0.037) and FSL (75 ± 31 cc; *p*<0.001) were both less accurate, but the mean absolute differences were smaller for SPM (31 ± 26 cc; 8%) than for FSL (75 ± 31 cc; 20%).

#### CSF volume

FSL showed accurate CSF measurements (Table 3 and the Bland Altman plots in Fig 4). The mean absolute difference was also smaller for FSL (42 ± 23 cc; 10%) than for the other methods. Both SPM (-80 ± 77 cc; *p* = 0.001) and Freesurfer (120 ± 68 cc; *p*<0.001) were less accurate for CSF volume (see also the Bland Altman plots in Fig 4) and had large mean absolute differences: SPM (95 ± 56 cc; 24%); Freesurfer (120 ± 68 cc; 30%).

#### ICV

FSL was accurate for ICV (Table 3 and the Bland Altman plots in Fig 4). The mean absolute difference was also smaller for FSL (39 ± 21 cc; 3%) than for the other methods. Both SPM (-78 ± 63 cc; *p*<0.001) and Freesurfer (70 ± 55 cc; *p*<0.001) were less accurate for ICV (also see the Bland Altman plots in Fig 4) and had large mean absolute differences: Freesurfer (74 ± 50 cc; 5%); SPM (84 ± 55 cc; 6%).

### Summary

A summary of the results of the robustness across field strengths as well as the accuracy analysis can be found in Table 4.

**Table 4:** Summary of robustness across field strength and accuracy results.

	TBV		GM		WM		CSF		ICV	
	*R*	*A*	*R*	*A*	*R*	*A*	*R*	*A*	*R*	*A*
**SPM**	4%	3%	5%	7%	4%	8%	14%	24%	2%	6%
**Freesurfer**	<1%	5%	1%	13%	2%	7%	34%	30%	9%	5%
**FSL**	1%	3%	2%	9%	3%	20%	10%	10%	3%	3%

Note: R: robustness, shown as the mean absolute difference between 3T and 1.5T measurements as a percentage of the mean measurement at 3T; A: accuracy, shown as the mean absolute difference between automated and manual measurements as a percentage of the mean manual measurement.

## Discussion

Brain volumes and ICV could be measured quite robustly in scans acquired using different MRI acquisition techniques. However, performance of SPM, Freesurfer and FSL varied depending on the assessed compartment.

### Comparison with previous studies

Few studies have evaluated the robustness across different field strengths of brain volume and ICV measurements. Previous work has focused on ICV measurements with older software versions of SPM, Freesurfer and/or FSL. [4,5] One study assessed robustness of ICV measurements across field strengths using SPM5 and the Brain Extraction Tool (BET) of FSL and compared it with their own method. [5] This study showed that especially SPM5 and to a lesser extent BET showed large differences between ICV measurements at 3T and 1.5T. Another study focused on ICV measurements across field strengths obtained with Freesurfer. [4] This study showed that, using Freesurfer, systematic bias occurred in ICV measurements between 3T and 1.5T. The findings of both studies are in line with our study, showing that bias can occur in ICV measurements between 3T and 1.5T MRI data, especially when using Freesurfer. This might be caused by Freesurfer’s registration procedure, which is susceptible to (slight) differences in MRI acquisition techniques. Contrary to a previous study, SPM did show robust ICV measurements in our study. [5] This could be due to recent improvements in the segmentation algorithm (tissue classification, bias correction and image registration in the same generative model). The suboptimal performance of Freesurfer for ICV assessment is clearly an important issue. Correction for inter-subject variation in head size by using ICV is common practice in studies of brain volume and brain atrophy. [31] Hence, bias in ICV thus also affects brain volume analyses [32] To avoid this, a segmentation method should be chosen that has a robust ICV segmentation. Since none of the methods in our current study was robust as well as accurate for all volumes, it may be feasible to combine measurements obtained with different methods to get both robust and accurate brain volume and ICV measurements. As for robustness across spatial resolutions, we found similar results than two previous studies. [4,33] These studies, that only investigated the performance of (older versions of) Freesurfer, showed differences in spatial resolution could lead to variations in brain volume measurements. For an detailed overview of previous studies on robustness of brain volumes and other brain MRI abnormalities, specifically in the context of ageing related cerebrovascular disease, we refer to recently published work. [34]

### Strengths and limitations

The strength of our study is the set of high quality scan-rescan data, the selection of subjects (comparable with subjects in brain ageing studies, but without a primary cerebral disease) and the large number of manually segmented scans that allowed us to make a reliable comparison of the performance of the brain segmentation methods. In addition, our study is the first that assessed the robustness across different MRI acquisition techniques as well as accuracy of the most recent versions of three widely used automated methods for brain volume measurements in a common framework.

A limitation could be that manual segmentations were performed on MRI slices with a thickness of 3 mm. Although manual segmentations of higher resolution data might be preferable (i.e. with a slice thickness of 1 mm), creating these manual segmentations is very labor intensive. By selecting a lower resolution we chose to invest in a higher quantity of datasets to better include variations in brain anatomy. Importantly, our results were similar for non-down sampled 3D T1 images. Another limitation could be the relatively small sample size. However, we chose to invest in a high quality dataset that could assess both robustness and accuracy.

As is common in brain segmentation studies, we have compared binary manual segmentations with probabilistic (partial volume estimated) automated segmentations. Another approach could be the creation of probabilistic manual segmentations (e.g. by combining binary manual segmentations of the same subject but performed by different raters into a single probabilistic segmentation [35]. However, this is very labor intensive and has limited added value compared with manually segmenting more subjects.

Another limitation could be that variations in scanner related parameters might give differences in the measures of robustness of the different methods. Therefore, MRI data acquired with scanner parameters that are different from the ones we have used could possibly lead to a different ranking in performance of the methods for one or more of the tissue compartments considered. Moreover, presence of severe brain abnormalities (for example as seen in patients with dementia or multiple sclerosis) could potentially also lead to a different ranking in performance of the methods, as some methods might be more robust for brain abnormalities. Generalizability of our results should therefore be performed with caution.

### Conclusions

We showed that robust brain volume measurements can be obtained with state-of-the-art generic brain MRI analysis packages in datasets with different MRI acquisitions (such as different field strengths). However, all methods showed variations in robustness and accuracy over various tissue compartments. This needs to be taken into account when selecting an appropriate method in multicenter brain imaging studies.

## Supporting Information

S1 Dataset Robustness analysis data. TBV: total brain volume. GM: gray matter volume. WM: white matter volume. CSF: cerebrospinal fluid volume. ICV: intracranial volume.
(XLSX)

S2 Dataset Accuracy analysis data. TBV: total brain volume. SGM: supratentorial gray matter volume. SWM: supratentorial white matter volume. CSF: cerebrospinal fluid volume. ICV: intracranial volume. DSC: Dice’s similarity coefficient.
(XLSX)

S1 Fig Individual automated volume measurements 3T and 1.5T. X-axis: subject number. Y-axis: individual brain volume measurements (in cc). TBV: total brain volume. GM: gray matter volume. WM: white matter volume. CSF: cerebrospinal fluid volume. ICV: intracranial volume.
(TIF)

S2 Fig Individual automated volume measurements high and low spatial resolution. X-axis: subject number. Y-axis: individual brain volume measurements (in cc). TBV: total brain volume. GM: gray matter volume. WM: white matter volume. CSF: cerebrospinal fluid volume. ICV: intracranial volume.
(TIF)

S3 Fig Bland-Altman plots high and low spatial resolution. X-axis: mean brain volume measurement at high and low spatial resolution. Y-axis: difference (in cc) in brain volume measurement between high and low spatial resolution. The mean, lower (- 1.96 SD) and upper (+ 1.96 SD) limits of agreement are shown. A negative difference on the y-axis is seen when brain volume measurement at a lower resolution was larger than at a higher resolution. TBV: total brain volume. GM: gray matter volume. WM: white matter volume. CSF: cerebrospinal fluid volume. ICV: intracranial volume.
(TIF)

S4 Fig Individual automated and manual TBV, GM and WM measurements. TBV: total brain volume. GM: supratentorial gray matter volume. WM: supratentorial white matter volume.
(TIF)

S5 Fig Individual automated and manual CSF and ICV measurements. CSF: total cerebrospinal fluid volume. ICV: intracranial volume.
(TIF)

S1 Table Automated volume measurements across different spatial resolutions (n = 10). All volumes are expressed as means (in cc) ± SD. TBV: total brain volume. GM: gray matter volume. WM: white matter volume. CSF: cerebrospinal fluid volume. ICV: intracranial volume. T: Tesla.
(DOCX)

S2 Table Robustness analysis across different spatial resolutions (n = 10). All volumes (in cc) are expressed as means ± SD. Coefficients of repeatability are expressed as a volume (in cc). TBV: total brain volume. GM: gray matter volume. WM: white matter volume. CSF: cerebrospinal fluid volume. ICV: intracranial volume. T: Tesla. Mean differences between high and low resolutions were tested for each method separately using Wilcoxon signed rank test (* p<0.05).
(DOCX)
