# Supplementary figures and images for: Robustness of Automated Methods for Brain Volume Measurements across Different MRI Field Strengths
Source: PLoS One. 2016 Oct 31;11(10):e0165719. doi: 10.1371/journal.pone.0165719 (PMC5087903; doi:10.1371/journal.pone.0165719)

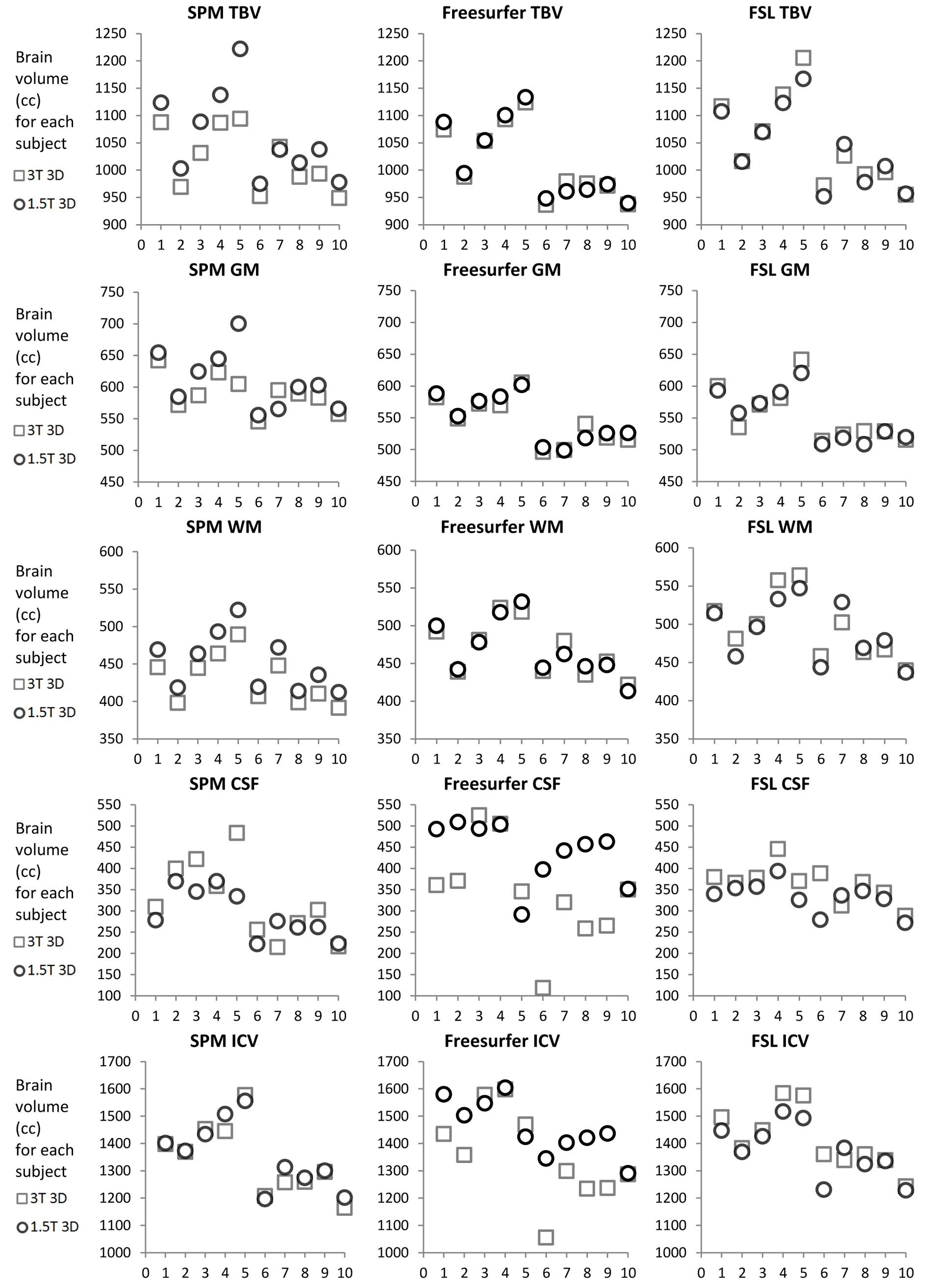

Supplement: S1 Fig — X-axis: subject number. Y-axis: individual brain volume measurements (in cc). TBV: total brain volume. GM: gray matter volume. WM: white matter volume. CSF: cerebrospinal fluid volume. ICV: intracranial volume. (TIF) [file pone.0165719.s003.tif]

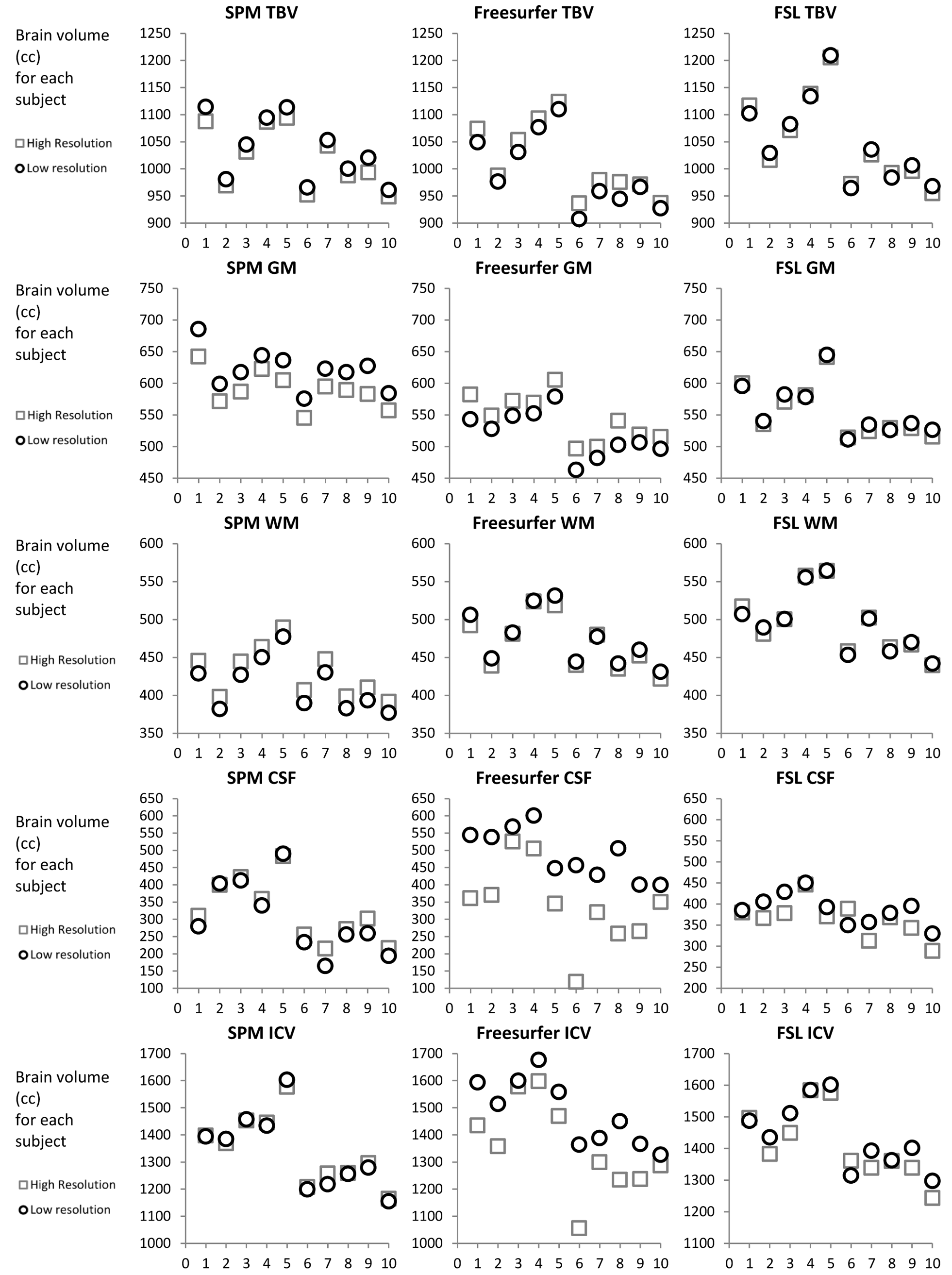

Supplement: S2 Fig — X-axis: subject number. Y-axis: individual brain volume measurements (in cc). TBV: total brain volume. GM: gray matter volume. WM: white matter volume. CSF: cerebrospinal fluid volume. ICV: intracranial volume. (TIF) [file pone.0165719.s004.tif]

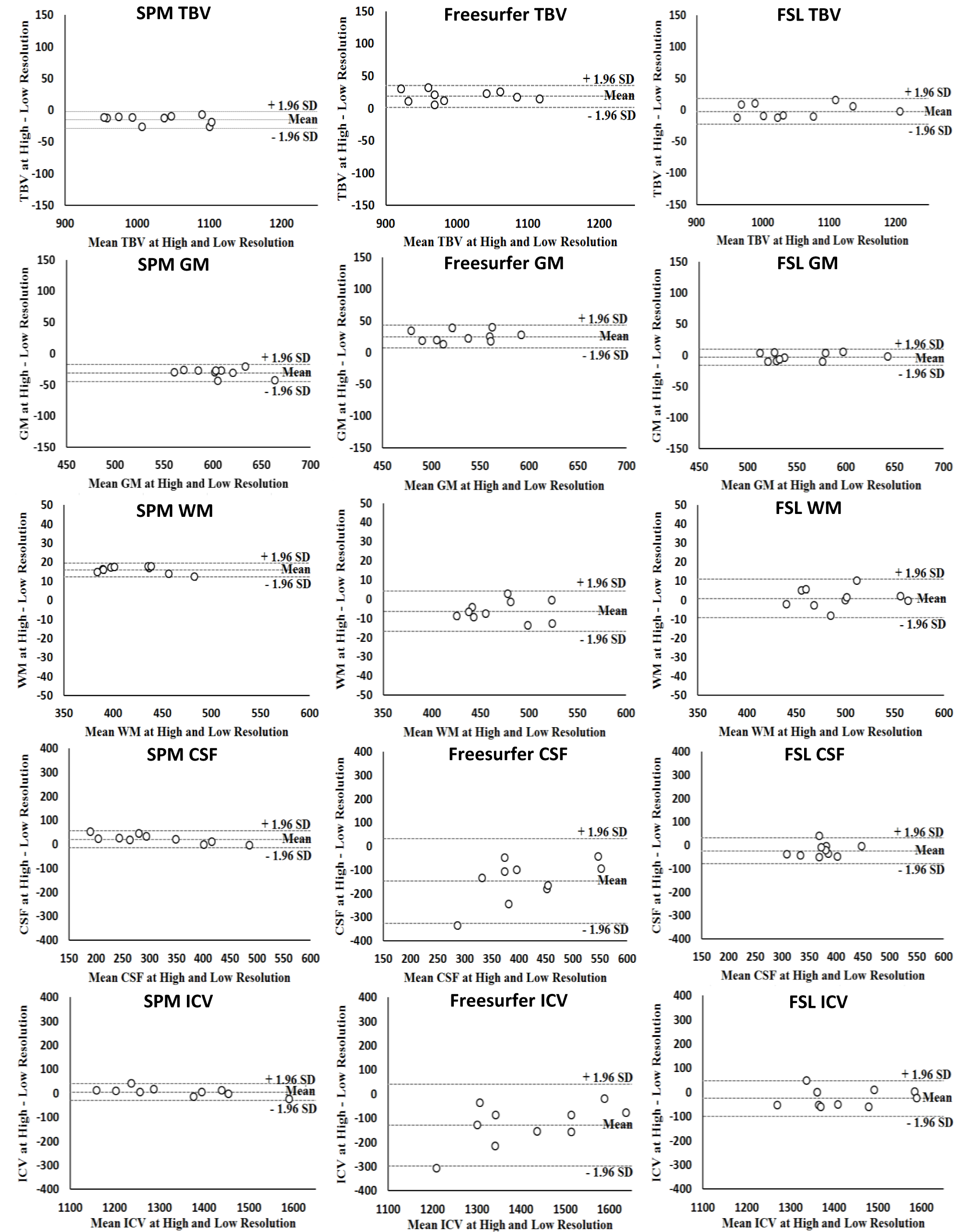

Supplement: S3 Fig — X-axis: mean brain volume measurement at high and low spatial resolution. Y-axis: difference (in cc) in brain volume measurement between high and low spatial resolution. The mean, lower (- 1.96 SD) and upper (+ 1.96 SD) limits of agreement are shown. A negative difference on the y-axis is seen when brain volume measurement at a lower resolution was larger than at a higher resolution. TBV: total brain volume. GM: gray matter volume. WM: white matter volume. CSF: cerebrospinal fluid volume. ICV: intracranial volume. (TIF) [file pone.0165719.s005.tif]

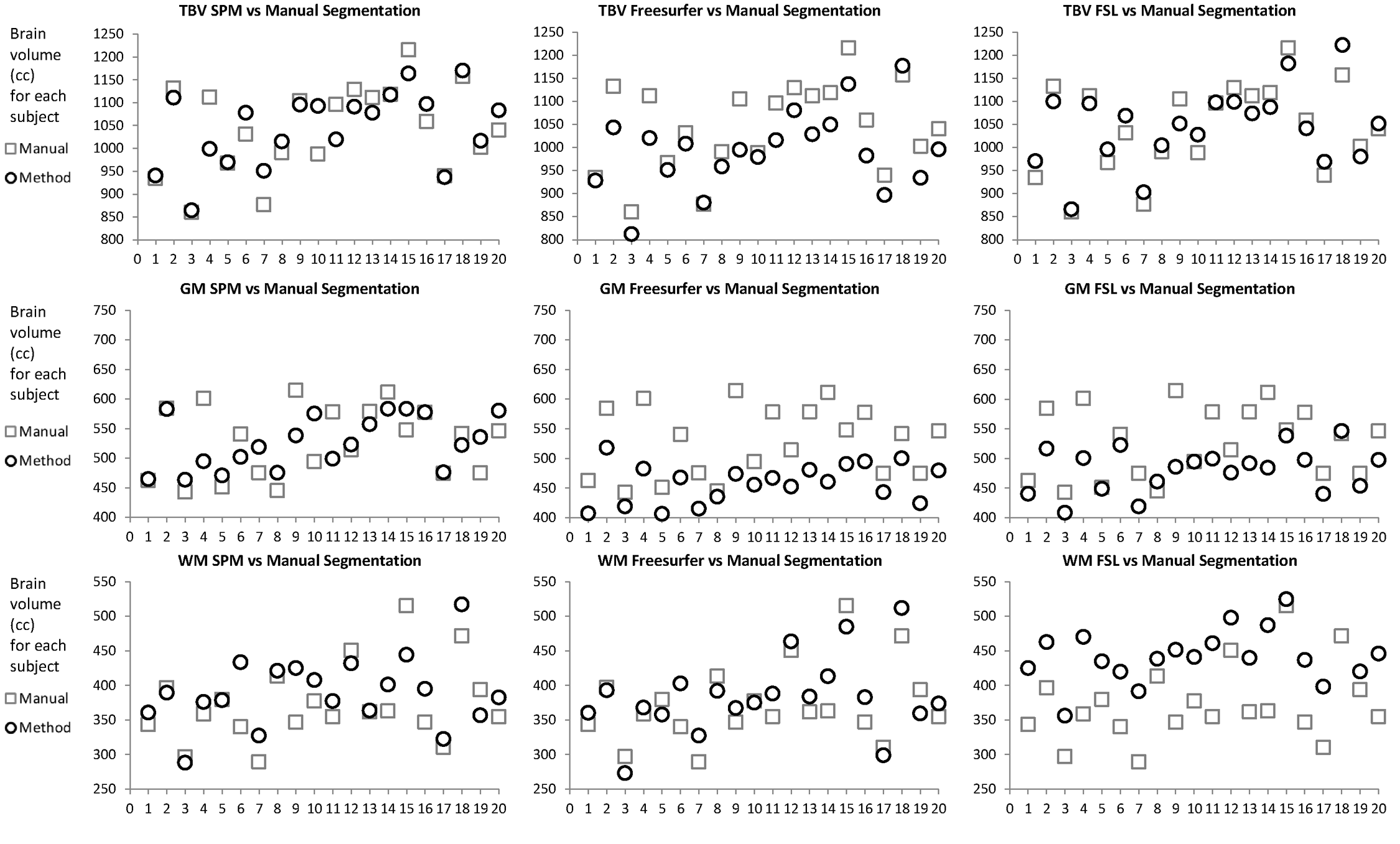

Supplement: S4 Fig — TBV: total brain volume. GM: supratentorial gray matter volume. WM: supratentorial white matter volume. (TIF) [file pone.0165719.s006.tif]

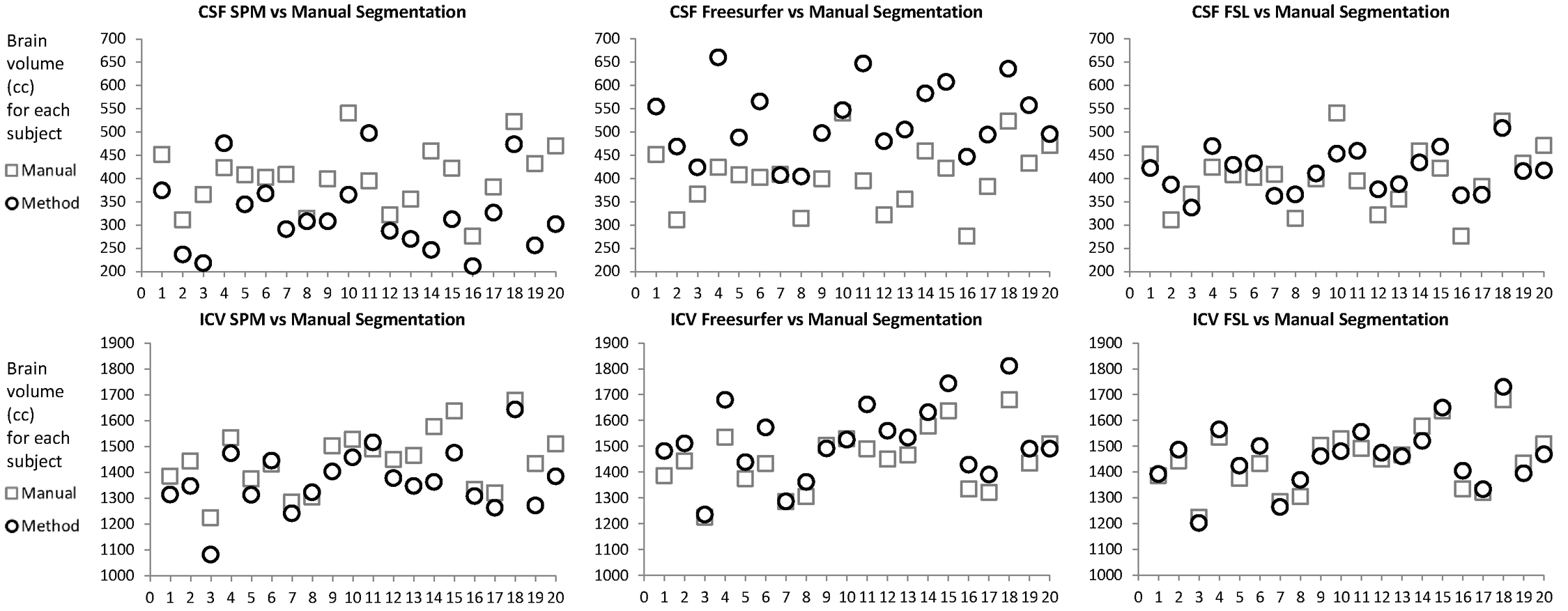

Supplement: S5 Fig — CSF: total cerebrospinal fluid volume. ICV: intracranial volume. (TIF) [file pone.0165719.s007.tif]
