# Supplementary material for: Robustness of Automated Methods for Brain Volume Measurements across Different MRI Field Strengths
Source: PLoS One. 2016 Oct 31;11(10):e0165719. doi: 10.1371/journal.pone.0165719 (PMC5087903; doi:10.1371/journal.pone.0165719)
Supplement: S1 Table — All volumes are expressed as means (in cc) ± SD. TBV: total brain volume. GM: gray matter volume. WM: white matter volume. CSF: cerebrospinal fluid volume. ICV: intracranial volume. T: Tesla. (DOCX) [file pone.0165719.s008.docx]

|  | **Resolution** | | **SPM** | **Freesurfer** | **FSL** |
| --- | --- | --- | --- | --- | --- |
| **TBV** | *3T* | *Low* | 1035 ± 59 | 995 ± 68 | 1052 ± 80 |
|  |  | *High* | 1020 ± 57 | 1013 ± 67 | 1049 ± 82 |
| **GM** | *3T* | *Low* | 621 ± 32 | 520 ± 36 | 558 ± 42 |
|  |  | *High* | 590 ± 29 | 545 ± 37 | 554 ± 43 |
| **WM** | *3T* | *Low* | 414 ± 34 | 475 ± 36 | 494 ± 41 |
|  |  | *High* | 430 ± 33 | 468 ± 36 | 495 ± 42 |
| **CSF** | *3T* | *Low* | 303 ± 105 | 489 ± 72 | 387 ± 36 |
|  |  | *High* | 323 ± 90 | 342 ± 118 | 364 ± 43 |
| **ICV** | *3T* | *Low* | 1338 ± 140 | 1484 ± 121 | 1439 ± 106 |
|  |  | *High* | 1343 ± 128 | 1355 ± 168 | 1413 ± 111 |
