# Supplementary material for: Robustness of Automated Methods for Brain Volume Measurements across Different MRI Field Strengths
Source: PLoS One. 2016 Oct 31;11(10):e0165719. doi: 10.1371/journal.pone.0165719 (PMC5087903; doi:10.1371/journal.pone.0165719)
Supplement: S2 Table — All volumes (in cc) are expressed as means ± SD. Coefficients of repeatability are expressed as a volume (in cc). TBV: total brain volume. GM: gray matter volume. WM: white matter volume. CSF: cerebrospinal fluid volume. ICV: intracranial volume. T: Tesla. Mean differences between high and low resolutions were tested for each method separately using Wilcoxon signed rank test (* p<0.05). (DOCX) [file pone.0165719.s009.docx]

|  |  | | **SPM** | **Freesurfer** | **FSL** |
| --- | --- | --- | --- | --- | --- |
| **TBV** | *High vs low (3T)* | *Mean difference* | -15 ± 7^*^ | 18 ± 9^*^ | -3 ± 11 |
|  |  | *Mean absolute difference* | 15 ± 7 | 18 ± 9 | 10 ± 4 |
|  |  | *as % of mean TBV at 3D* | 2 | 2 | 1 |
|  |  | *Coefficient of repeatability* | 33 | 40 | 21 |
|  |  | *as % of mean TBV at 3D* | 3 | 4 | 2 |
| **GM** | *High vs low (3T)* | *Mean difference* | -31 ± 7^*^ | 25 ± 9^*^ | -3 ± 7 |
|  |  | *Mean absolute difference* | 31 ± 7 | 25 ± 9 | 6 ± 4 |
|  |  | *as % of mean GM at 3D* | 5 | 5 | 1 |
|  |  | *Coefficient of repeatability* | 64 | 53 | 14 |
|  |  | *as % of mean GM at 3D* | 11 | 10 | 3 |
| **WM** | *High vs low (3T)* | *Mean difference* | 16 ± 2^*^ | -6 ± 5^*^ | <1 ± 5 |
|  |  | *Mean absolute difference* | 16 ± 2 | 7 ± 5 | 4 ± 3 |
|  |  | *as % of mean WM at 3D* | 4 | 1 | 1 |
|  |  | *Coefficient of repeatability* | 32 | 16 | 10 |
|  |  | *as % of mean WM at 3D* | 7 | 4 | 2 |
| **CSF** | *High vs low (3T)* | *Mean difference* | 20 ± 18 | -147 ± 91^*^ | -23 ± 29^*^ |
|  |  | *Mean absolute difference* | 22 ± 15 | 147 ± 91 | 31 ± 19 |
|  |  | *as % of mean CSF at 3D* | 7 | 43 | 9 |
|  |  | *Coefficient of repeatability* | 53 | 341 | 71 |
|  |  | *as % of mean CSF at 3D* | 16 | 99.8 | 20 |
| **ICV** | *High vs low (3T)* | *Mean difference* | 5 ± 18 | -129 ± 86^*^ | -26 ± 38^*^ |
|  |  | *Mean absolute difference* | 14 ± 12 | 129 ± 86 | 37 ± 25 |
|  |  | *as % of mean ICV at 3D* | 1 | 9 | 3 |
|  |  | *Coefficient of repeatability* | 35 | 305 | 88 |
|  |  | *as % of mean ICV at 3D* | 3 | 23 | 6 |
